# Epidemiological characteristics of cervical cancer in Asian populations and risk factor inference based on Mendelian randomization

**DOI:** 10.1097/MD.0000000000051015

**Published:** 2026-10-02

**Authors:** Bing Wu, Qin Liu

**Affiliations:** a Department of Gynecology, Affiliated Kunshan Hospital of Jiangsu University, Jiangsu University, Suzhou, Jiangsu, China.

**Keywords:** Asian populations, cervical cancer, disease burden, Mendelian randomization

## Abstract

This study aimed to systematically investigate the epidemiological distribution characteristics and disease burden of cervical cancer (CC) in Asian populations, and to assess the causal relationship between smoking, a key modifiable risk factor, and the incidence of CC based on genetic evidence, thereby providing a scientific basis for targeted prevention and control strategies in Asia. This study first used the GBD 2021 database to analyze trends in CC incidence, prevalence, mortality, and disability-adjusted life years (DALYs) from 1990 to 2021 across global, Asian, and East Asian regions, along with age distribution and attributable risk factors. It then applied 2-sample Mendelian randomization (MR) using genome-wide association study (GWAS) data from East Asian populations, with multiple smoking-related behaviors (e.g., current smoking, cigarettes per day, household smoking exposure) as exposures and CC as the outcome. GBD analysis revealed that in 2021, Asia accounted for over half of the global CC disease burden. Although point estimates suggested a potential upward trend in CC burden across Asia from 1990 to 2021, statistical significance was not reached for the overall Asian population. Despite a general decline in the global incidence rate, the incidence in Japan exhibited a statistically supported upward trend, while point estimates in several other East Asian countries also suggested an increase. The peak disease burden was concentrated among women aged 55 to 59 years. Risk attribution analysis indicated that unsafe sexual behavior was the primary attributable factor for CC-related DALYs, while the attributable burden of smoking was significantly higher in Japan than in other regions. However, MR analysis did not identify statistically significant causal associations between any smoking-related exposure factors and CC risk (*P*-FDR ≥ .05 after false discovery rate correction). Sensitivity analyses revealed no substantial heterogeneity or pleiotropy bias. Asia, particularly East Asia, faces a substantial and potentially growing burden of CC. Causal inference based on genetic data from East Asian populations did not confirm a direct causal relationship between smoking and the incidence of cervical cancer. Future studies with larger genetic samples are needed to further elucidate the precise role of smoking.

## 1. Introduction

Cervical cancer (CC) is the fourth most commonly diagnosed malignant tumor among women worldwide, and it is one of the leading causes of cancer-related deaths in women globally, with approximately 6,60,000 new cases reported in 2022.^[1,2]^ According to the World Health Organization’s Global Strategy for CC Elimination, which defines the elimination of CC as an annual incidence rate of fewer than 4 cases per 1,00,000 women, it is estimated that approximately 3,50,000 women globally, including 44,750 women in China, are projected to avoid death each year.^[3]^ Despite a significant decline in the incidence of CC over the past 3 decades, it remains a global public health issue with notable regional disparities in both incidence and mortality rates.^[4]^ With restricted resources and inadequate interventions, the lion’s share of the disease burden falls on low- and middle-income countries, which make up 80% of all CC cases.^[5]^ CC incidence and mortality peak in regions such as Africa, Melanesia, South America, Southeast Asia, and Central-South Asia, with age-standardized incidence at 15.3 to 40.1 per 1,00,000 women and mortality at 7.8 to 28.6 per 1,00,000 women.^[6,7]^ Although the incidence rate of CC in East Asia is 10.8 per 1,00,000 women, it remains at a relatively high level compared with that in developed countries such as Europe and the United States.^[8]^ Consequently, examining the distribution of CC in the Asian population aids in identifying the disparities in its epidemiological features compared to global trends, providing crucial insights and implications for targeted regional prevention and management.

Reportedly, other risk factors that lead to the development of CC include early sexual activity, having multiple sexual partners, and smoking.^[9]^ Nevertheless, marked differences remain, especially in low- and middle-income nations with constrained access to prevention and treatment services. Smoking serves as a key modifiable risk factor for CC by promoting the persistence of HPV infection and weakening immune defenses.^[9]^ Over 30,000 CC deaths in 2019 were linked to smoking, underscoring the significant influence of this modifiable risk factor.^[10]^ Furthermore, smoking is one of the 2 attribution analysis outcomes in the Global Burden of Disease (GBD) database contributing to disability-adjusted life years (DALYs) from CC, with the other being unsafe sexual behavior.^[11]^ However, the findings from these studies indicate an absence of research employing causal inference methods to investigate the link between smoking behaviors and CC occurrence in Asia.

Consequently, this study aims to utilize the GBD database to investigate the epidemiological trends of CC between 1990 and 2021 across Asia and East Asian nations, while also analyzing age-specific distributions and the main contributors to DALYs. Building on this foundation, we utilized genome-wide association study (GWAS) datasets from East Asia and applied Mendelian randomization (MR) methods to infer the causal relationship between smoking – a major factor contributing to DALYs – and the development of CC in the Asian population. By integrating GBD data and MR analysis, our study explores the distribution characteristics of CC among Asian women and the causal effect of smoking on CC incidence with more reliable methods, thereby enhancing the credibility of the findings.

## 2. Materials and methods

### 2.1. GBD data analysis

GBD 2021 stands as the latest version of this multinational collaborative research, conducting quantitative analysis of health losses attributable to a broader spectrum of pathological conditions and behavioral/environmental determinants across 204 geopolitical units and 21 subregional groups over a 32-year observation period from 1990 to 2021. This study covers 204 countries and territories between 1990 and 2021 and incorporates methodological innovations for adjusting pandemic-era data.^[12]^ CC is diagnosed and classified according to the ICD-10 coding system. To capture data uncertainty, a 95% uncertainty interval (UI) is used to indicate the reliability range of study estimates. Data on CC metrics (incidence, prevalence, mortality, and DALYs) were obtained from the IHME’s (Institute for Health Metrics and Evaluation) Global Health Data Exchange platform (GHDx, https://www.healthdata.org/). Crude rates were used to describe the observed burden, whereas age-standardized rates (ASRs) were used for comparisons across populations and years with different age structures. Temporal trends were assessed using the estimated annual percentage change (EAPC). An increase or decrease was considered statistically supported only when the 95% UI of the EAPC did not include 0. The Asian population was analyzed with age-stratified methods, and the main contributors to DALYs in this region were subsequently evaluated.

Focusing on East Asian nations – driven by the use of East Asian (Japanese) GWAS data in the follow-up MR analysis – this study examined CC changes across global, Asian, and East Asian (GBD-defined: China, North Korea, China Taiwan) aggregates, as well as individual entities like China, North Korea, Japan, and South Korea.

### 2.2. MR study design

According to the attribution analysis in Section 2.1, unsafe sexual practices and tobacco use were identified as key contributors to DALYs associated with CC. Given the established association between unsafe sexual behavior and CC, as well as the scarcity of GWAS datasets relevant to Asian populations, this study applied 2-sample MR to examine potential causal links between smoking-related behaviors (current tobacco smoking, cigarettes per day, smoking/smokers in household, exposure to tobacco smoke at home, exposure to tobacco smoke outside home) and CC. In line with MR assumptions, independent single nucleotide polymorphisms (SNPs) were utilized as instrumental variables (IVs), guided by 3 fundamental principles^[13]^: the IVs must show robust associations with the exposure; the IVs must be independent of potential confounding factors; and the IVs must affect the outcome exclusively via the exposure.

In this study, all genome-wide association analysis data were obtained from publicly shared East Asian (Japanese) datasets available through OpenGWAS (https://opengwas.io/datasets/). Ethical approval was not needed as consent procedures had been completed in the original studies.

### 2.3. Data sources for MR analysis

All GWAS datasets used in this study were restricted to individuals of East Asian ancestry. CC (Open GWAS ID: ebi-a-GCST90018597) comprised 61,581 East Asian participants, including 967 CC cases, with 1,24,13,631 SNPs. Current tobacco smoking (Open GWAS ID: ukb-e-1239_EAS) included 2662 East Asian participants and 82,56,004 SNPs. Cigarettes per day (ieu-b-5071) involved 72,655 East Asian participants and 3,10,89,300 SNPs. Smoking/smokers in household (Open GWAS ID: ukb-e-1259_EAS) contained 2498 East Asian participants and 82,41,178 SNPs. Exposure to tobacco smoke at home (Open GWAS ID: ukb-e-1269_EAS) consisted of 2312 East Asian participants and 82,48,108 SNPs. Exposure to tobacco smoke outside home (Open GWAS ID: ukb-e-1279_EAS) included 2006 East Asian participants and 82,59,212 SNPs.

### 2.4. IVs selection and MR analysis

The criteria for screening IVs were established as follows: SNPs were selected as IVs under a liberal genome-wide significance threshold (*P* < 1 × 10^-6^) to ensure an adequate number of SNPs for MR analysis, followed by clumping to remove linkage disequilibrium (parameters: *r*^2^ = 0.001, region length = 10,000 kb). The strength of the selected IVs was evaluated using the *F*-statistic (calculated as *F* = *R*^2^(N-2)/(1-*R*^2^), where higher values indicate stronger instrument strength; SNPs with an *F*-statistic below 10 were excluded to minimize weak instrument bias^[14]^; The LDtrait online tool (https://ldlink.nih.gov/) was utilized to examine the phenotypic associations of the SNPs, ensuring that their influence on the outcome was exclusively mediated through the exposure.^[15]^ SNPs associated with potential confounders (e.g., HPV infection) were identified and removed via LDtrait online tool.

Two-sample MR analyses were conducted using 5 approaches: MR-Egger, inverse-variance weighted (IVW), weighted median, weighted mode, and simple mode, with the IVW method serving as the primary reference. SNPs were employed as instrumental variables to assess the causal relationship between exposures and CC. Sensitivity analyses were performed to evaluate the robustness of the results against potential violations of MR assumptions. Heterogeneity among SNPs was assessed using Cochran *Q* test. Potential horizontal pleiotropy was examined through MR-Egger regression and the MR-Pleiotropy Residual Sum and Outlier (MR-PRESSO) global test. A statistically significant intercept (*P* < .05) in the MR-Egger regression indicated the presence of horizontal pleiotropy.^[15]^ MR-PRESSO was applied to detect outlier SNPs, and all MR analyses were repeated after removing these outliers. Additionally, a leave-one-out sensitivity analysis was conducted to evaluate the influence of individual SNPs on the causal estimates. All 2-sample MR analyses were performed using R software (version 4.3.1) with the “TwoSampleMR” package and the “MR-PRESSO” package. To reduce the likelihood of false-positive findings, false discovery rate (FDR) correction was applied, with *P*-FDR < .05 considered indicative of a significant causal relationship between exposure and outcome.

## 3. Results

### 3.1. Epidemiological characteristics of CC in the Asian population

In 2021, the global number of prevalent CC cases was 3.3845 million (95% UI: 3.1084–3.6973 million), with a prevalence rate of 79.30 (95% UI: 72.81–86.58) per 1,00,000 population. Compared with 1990, the global EAPC for prevalence was 1.48% (95% UI: −8.26, 11.89), and the 95% UI included 0, indicating no statistically supported temporal change. In 2021, Asia accounted for 53.47% (180.96 × 10^4^/ 338.45 × 10^4^) of global absolute prevalent CC cases, with an age-standardized prevalence rate of 69.70 (95% UI: 60.94, 79.52) per 1,00,000 population. The EAPC for prevalence in Asia was 11.00% (95% UI: −5.38 to 31.44). Since the 95% UI included 0, there was no statistically significant temporal change, although the point estimate suggested a potential upward trend in CC burden. In East Asia, the EAPC for prevalence was 67.9% (95% UI: 10.15, 134.03), indicating a statistically significant upward trend. Further analysis of CC prevalence trends in East Asian countries and regions – namely China, North Korea, South Korea, Mongolia, and Japan – revealed that South Korea had a negative point-estimate EAPC for prevalence (−25.39%, 95% UI: −43.28%, 2.42%); however, because the 95% UI included 0, a statistically supported decline could not be confirmed (Table 1).

**Table 1:** Prevalence and incidence of cervical cancer in 2021 and changes from 1990 to 2021.

Location	Prevalence (95% UI)			Incidence (95% UI)		
	No, 10^4^	ASR per 1,00,000	EAPC	No, 10^4^	ASR per 1,00,000	EAPC
Global	338.45 (310.84, 369.73)	79.30 (72.81, 86.58)	1.48 (−8.26, 11.89)	66.74 (61.30, 72.64)	15.32 (14.08, 16.68)	−15.44 (−23.27, −7.07)
Asia	180.96 (158.07, 206.72)	69.70 (60.94, 79.52)	11.00 (−5.38, 31.44)	35.77 (31.42, 40.32)	13.63 (11.98,15.36)	−16.21 (−28.4, −1.69)
East Asia	77.47 (56.17, 100.99)	79.16 (57.42, 102.7)	67.9 (10.15, 134.03)	13.79 (10.11, 17.78)	13.4 (9.86, 17.38)	10.19 (−27.78, 51.21)
China	74.65 (53.29, 98.31)	79.03 (56.34, 103.91)	77.12 (12.89, 149.45)	13.28 (9.60, 17.26)	13.37 (9.61, 17.51)	13.29 (−27.67, 57.71)
North Korea	1.59 (1.03, 2.22)	94.85 (61.31, 133.7)	43.93 (−15.05, 128.60)	0.30 (0.21, 0.43)	17.45 (11.82, 24.70)	21.31 (−23.98, 88.96)
Mongolia	0.14 (0.10, 0.18)	79.12 (58.55, 103.35)	8.73 (−21.57, 49.21)	0.03 (0.02, 0.04)	18.35 (13.61, 24.61)	−13.96 (−38.42, 13.97)
Japan	7.39 (6.84, 7.90)	89.78 (83.79, 95.97)	34.47 (25.31, 44.87)	1.18 (1.07, 1.26)	12.35 (11.56, 13.11)	15.00 (7.68, 22.22)
South Korea	2.17 (1.71, 2.83)	61.26 (48.64, 79.26)	−25.39 (−43.28, 2.42)	0.34 (0.27, 0.45)	8.88 (7.12, 11.47)	−42.18 (−55.47, −19.6)

ASR = age-standardized rate, EAPC = estimated annual percentage change, UI = uncertainty interval.

Regarding incidence, the global CC incidence was 66.74 (95% UI: 61.30, 72.64) per 1,00,000 population, with an ASR of 15.32 (95% UI: 14.08, 16.68). The EAPC from 1990 to 2021 showed a decrease of 15.44% (95% UI: −23.27, −7.07). Similarly, the EAPC for CC incidence in Asia also declined overall, at −16.21% (95% UI: −28.4, −1.69). At the regional level, however, the EAPC for CC incidence from 1990 to 2021 showed that point estimates suggested an upward trend in East Asia (10.19%, 95% UI: −27.78, 51.21), China (13.29%, 95% UI: −27.67, 57.71), and North Korea (21.31%, 95% UI: −23.98, 88.96), although statistical significance was not reached; only Japan showed a statistically supported increase (15.00%, 95% UI: 7.68, 22.22; Table 1).

The global EAPC for the age-standardized incidence rate was −15.44% (95% UI: −23.27% to −7.07%), indicating a statistically supported decline from 1990 to 2021. Asia also followed this overall downward trend, with an EAPC of −16.21% (95% UI: −28.4, −1.69). Regionally, the trend diverged, with Japan showing a statistically supported increase in incidence, while point estimates for several other East Asian entities suggested an upward trend (Table 2).

**Table 2:** Deaths and DALYs of cervical cancer in 2021 and changes from 1990 to 2021.

Location	Deaths (95% UI)			DALYs (95% UI)		
	No, 10^4^	ASR per 1,00,000	EAPC	No, 10^4^	ASR per 1,00,000	EAPC
Global	29.67 (27.21, 32.19)	6.62 (6.07, 7.18)	−31.61 (−37.98, −24.73)	991.17 (905.33, 1079.83)	226.28 (206.51, 246.86)	−31.45 (−38.14, −24.11)
Asia	16.07 (14.31, 17.94)	6.06 (5.39, 6.76)	−38.01 (−46.90, −27.34)	531.88 (473.32, 591.64)	201.31 (179.25, 223.75)	−39.52 (−48.12, −29.25)
East Asia	5.20 (3.94, 6.65)	4.68 (3.55, 5.98)	−34.11 (−56.16, −10.53)	161.62 (119.54, 208.01)	151.15 (111.8, 195.41)	−34.83 (−57.22, −10.95)
China	4.984 (3.69, 6.44)	4.64 (3.44, 6.00)	−33.47 (−56.72, −8.58)	154.78 (111.98, 200.92)	149.84 (108.94, 195.58)	−34.34 (−57.91, −8.85)
North Korea	0.01 (0.01, 0.02)	7.6 (5.30, 10.91)	−2.44 (−35.89, 46.73)	4.75 (3.22, 6.82)	268.44 (182.00, 385.08)	1.02 (−35.40, 53.48)
Mongolia	0.01 (0.01,0.02)	9.64 (7.14, 12.68)	−29.57 (−49.96, −6.40)	0.49 (0.36,0.65)	303.39 (224.72, 400.71)	−34.01 (−52.02, −13.74)
Japan	0.39 (0.33, 0.43)	2.74 (2.52, 2.88)	−24.55 (−26.89, −22.43)	10.03 (9.11, 10.61)	97.47 (92.46, 101.36)	−8.94 (−11.68, −6.30)
South Korea	0.11 (0.08, 0.14)	2.25 (1.77, 2.89)	−66.59 (−74.74, −54.36)	2.93 (2.30, 3.84)	70.79 (56.61, 91.26)	−69.22 (−76.96, −57.13)

ASR = age-standardized rate, DALYs = disability-adjusted life years, EAPC = estimated annual percentage change, UI = uncertainty interval.

Globally, CC accounted for 9.9117 million DALYs in 2021 (95% UI: 9.0533–10.7983 million), with an age-standardized DALY rate of 15.32 (95% UI: 14.08–16.68) per 1,00,000 population. From 1990 to 2021, the EAPC in DALYs showed a significant decrease of −31.45% (95% UI: −38.14, −24.11). A regional analysis revealed that while the EAPC declined across Asia, East Asia, and most other regions, North Korea was the sole exception, registering an increase of 1.02% (95% UI: −35.40, 53.48; Table 2).

### 3.2. Analysis of changes in age-standardized epidemiological indicators and risk factors in Asia

We analyzed the distribution of age-standardized prevalence, incidence, mortality, and DALYs among Asian women across different age groups. The results revealed that the total age-standardized prevalence of CC was highest among women aged 40 to 54, while the number of cases per 1,00,000 population remained elevated in the 40 to 59 age group (Fig. 1A). Both the total number of CC cases and the incidence per 1,00,000 population peaked in the 55 to 59 age group (Fig. 1B). The total age-standardized deaths were highest among women aged 55 to 59, and mortality per 100,000 population showed a gradual increase with advancing age (Fig. 1C). Age-standardized DALYs and DALYs per 1,00,000 population were also highest in the 55 to 59 age group (Fig. 1D).

**Figure 1.**
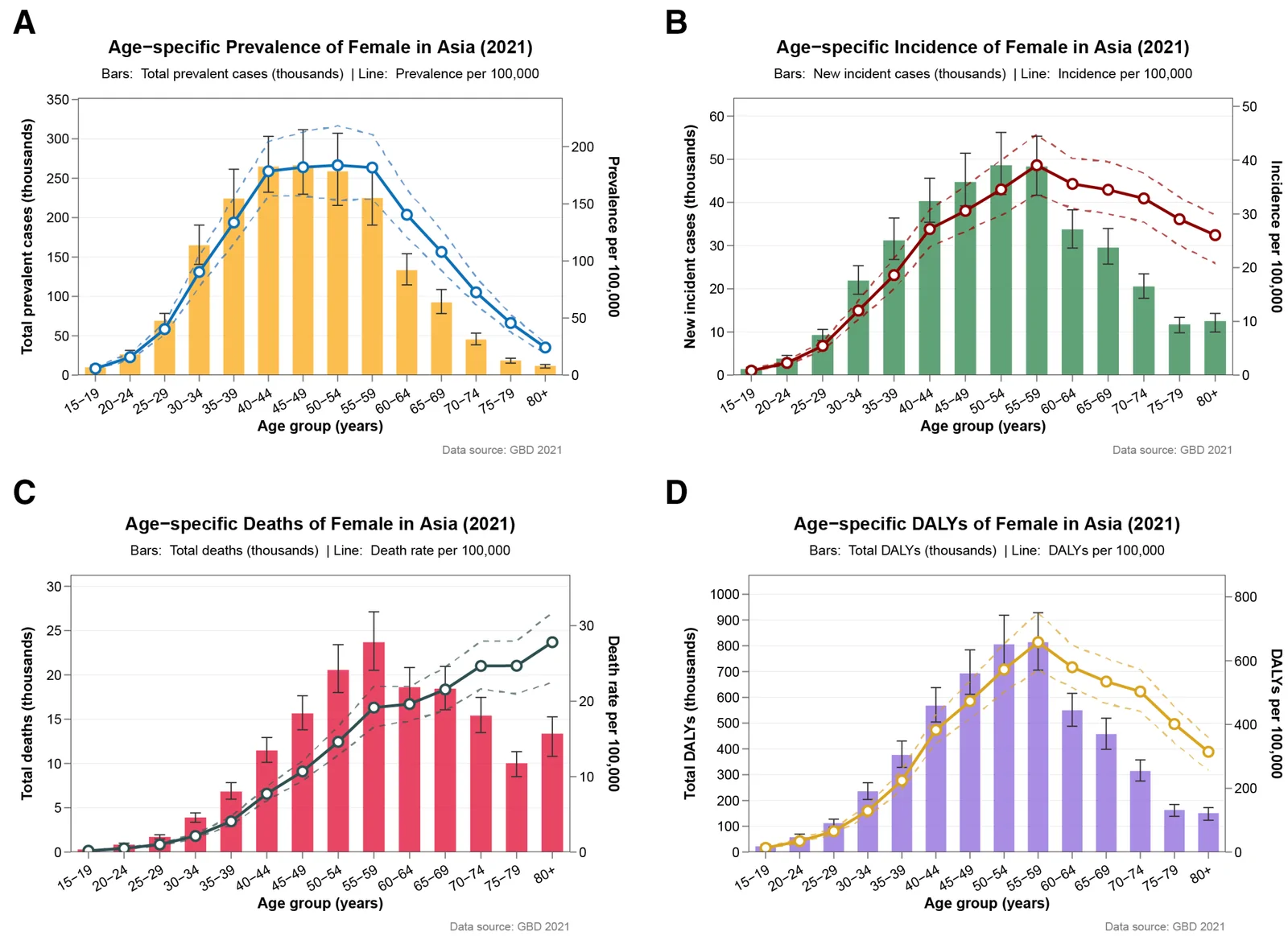
Age distribution characteristics of prevalence (A), incidence (B), deaths (C), and DALYs (D) among cervical cancer patients in Asia. DALYs = disability-adjusted life years.

Within the GBD attribution framework, unsafe sexual behavior accounted for 100% of the modeled attributable risk for CC-related DALYs globally and across the assessed Asian regions (Fig. 2A). Another identified risk factor was smoking. Smoking-attributable DALYs were highest in Japan (18.97%, 95% UI: 10.82–28.55), exceeding levels observed globally and in other East Asian countries and regions (Fig. 2B).

**Figure 2.**
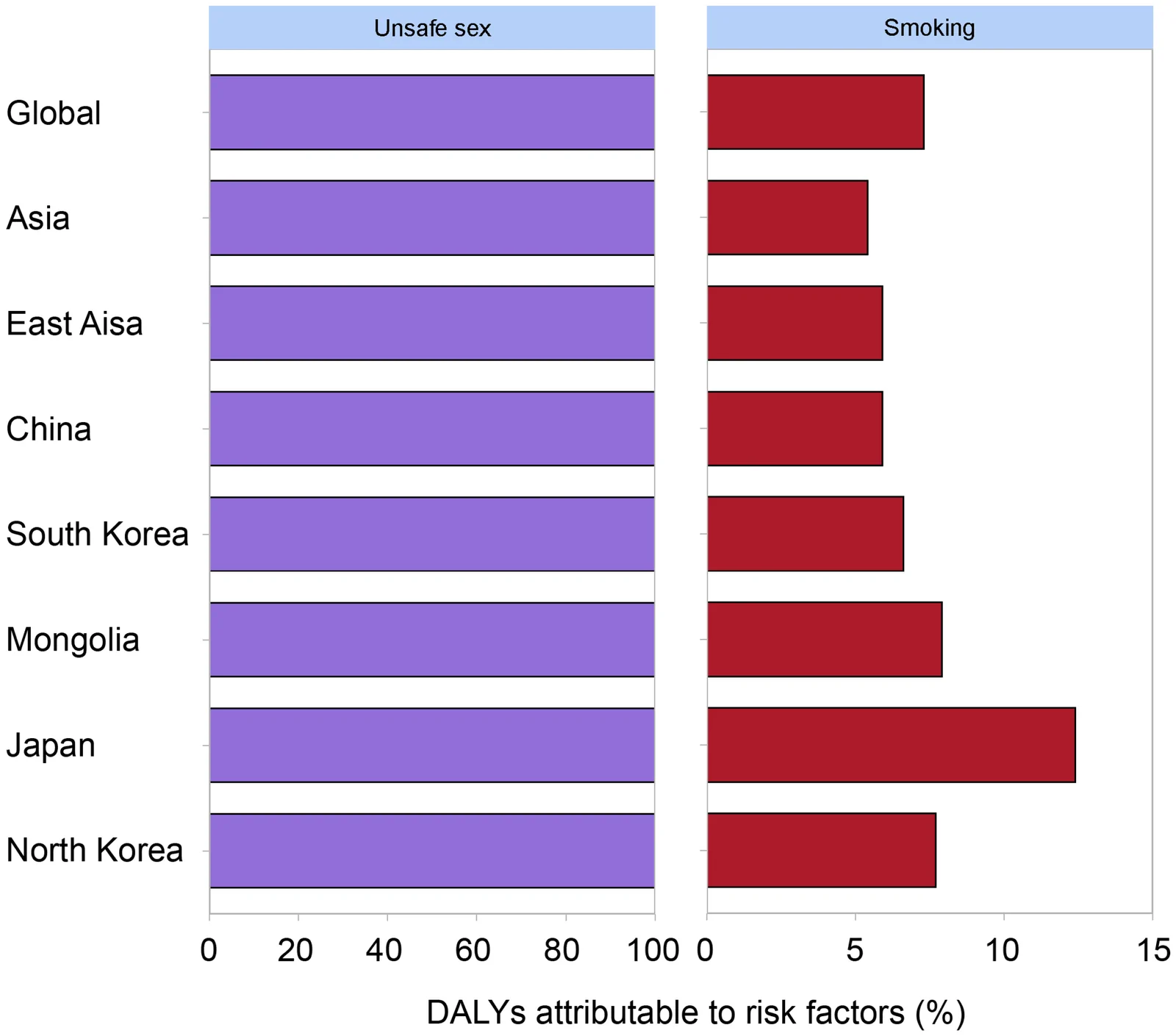
Attributable risk factors for cervical cancer DALYs in Asia: (A) Unsafe sex; (B) smoking. DALYs = disability-adjusted life years.

### 3.3. MR analysis of the causal relationship between smoking and CC

We performed MR analysis to investigate the causal relationships between CC and 5 tobacco-related exposure factors: current tobacco smoking, cigarettes per day, smoking/smokers in the household, exposure to tobacco smoke at home, and exposure to tobacco smoke outside the home. The results indicated that none of the 5 exposure factors showed a statistically significant causal association with CC (Table 3). Cochran *Q* test indicated no significant heterogeneity among the IVs used in the aforementioned 2-sample MR analysis. Furthermore, results from the MR intercept method and MR-PRESSO did not reveal significant horizontal pleiotropy among the IVs employed (Table 4 and Fig. 3A–E). Results from the leave-one-out sensitivity analysis demonstrated that the MR estimates were not driven by any single IV (Fig. 4A–E). Collectively, the suite of sensitivity analyses conducted affirms the reliability and robustness of the primary MR findings.

**Table 3:** Mendelian randomization analysis of the causal relationship between smoking and cervical cancer.

Exposure	Methods	IVs	β	OR	95% CI	*P*-value	*P*-FDR
Current tobacco smoking							
	MR-Egger	10	0.606	1.833	0.621–5.414	.305	.675
	Weighted median	10	−0.061	0.941	0.539–1.644	.832	.832
	IVW	10	0.135	1.145	0.742–1.766	.540	.675
	Simple mode	10	−0.307	0.736	0.341–1.589	.455	.675
	Weighted mode	10	−0.238	0.788	0.399–1.556	.510	.675
Cigarettes per day							
	MR-Egger	15	−0.680	0.507	0.166–1.550	.255	.557
	Weighted median	15	−0.401	0.669	0.291–1.539	.345	.557
	IVW	15	−0.180	0.836	0.459–1.521	.557	.557
	Simple mode	15	−0.560	0.571	0.126–2.583	.479	.557
	Weighted mode	15	−0.479	0.619	0.265–1.450	.288	.557
Smoking/smokers in household							
	MR-Egger	17	−1.012	0.364	0.109–1.217	.121	.607
	Weighted median	17	−0.016	0.984	0.529–1.831	.959	.959
	IVW	17	−0.059	0.943	0.596–1.492	.803	.959
	Simple mode	17	−0.301	0.740	0.277–1.977	.557	.959
	Weighted mode	17	−0.149	0.861	0.352–2.106	.748	.959
Exposure to tobacco smoke at home							
	MR-Egger	13	−0.401	0.670	0.302–1.485	.345	.784
	Weighted median	13	−0.165	0.848	0.487–1.478	.561	.784
	IVW	13	−0.174	0.840	0.537–1.314	.445	.784
	Simple mode	13	−0.115	1.122	0.497–2.536	.786	.784
	Weighted mode	13	−0.155	0.857	0.466–1.575	.627	0.784
Exposure to tobacco smoke outside home							
	MR-Egger	9	0.243	1.276	0.587–2.773	.558	.999
	Weighted median	9	0.026	1.026	0.712–1.479	.889	.999
	IVW	9	0.067	1.070	0.799–1.432	.650	.999
	Simple mode	9	0.046	1.047	0.680–1.611	.841	.999
	Weighted mode	9	0.000	1.000	0.643–1.556	.999	.999

CI = confidence interval, FDR = false discovery rate, IV = instrumental variable, IVW = inverse-variance weighted, OR = odds ratio.

**Table 4:** Sensitivity analysis of the Mendelian randomization study on smoking and cervical cancer.

	Cochran *Q* test		MR-Egger intercept	MR-PRESSO	MR-PRESSO global
	MR Egger	IVW			
Current tobacco smoking	0.264	0.269	0.379	0.555	0.298
Cigarettes per day	0.557	0.548	0.318	0.548	0.555
Smoking/smokers in household	0.936	0.840	0.115	0.761	0.854
Exposure to tobacco smoke at home	0.972	0.976	0.514	0.230	0.978
Exposure to tobacco smoke outside home	0.760	0.819	0.646	0.558	0.832

IVW = inverse-variance weighted, MR-PRESSO = MR Pleiotropy RESidual Sum and Outlier.

**Figure 3.**
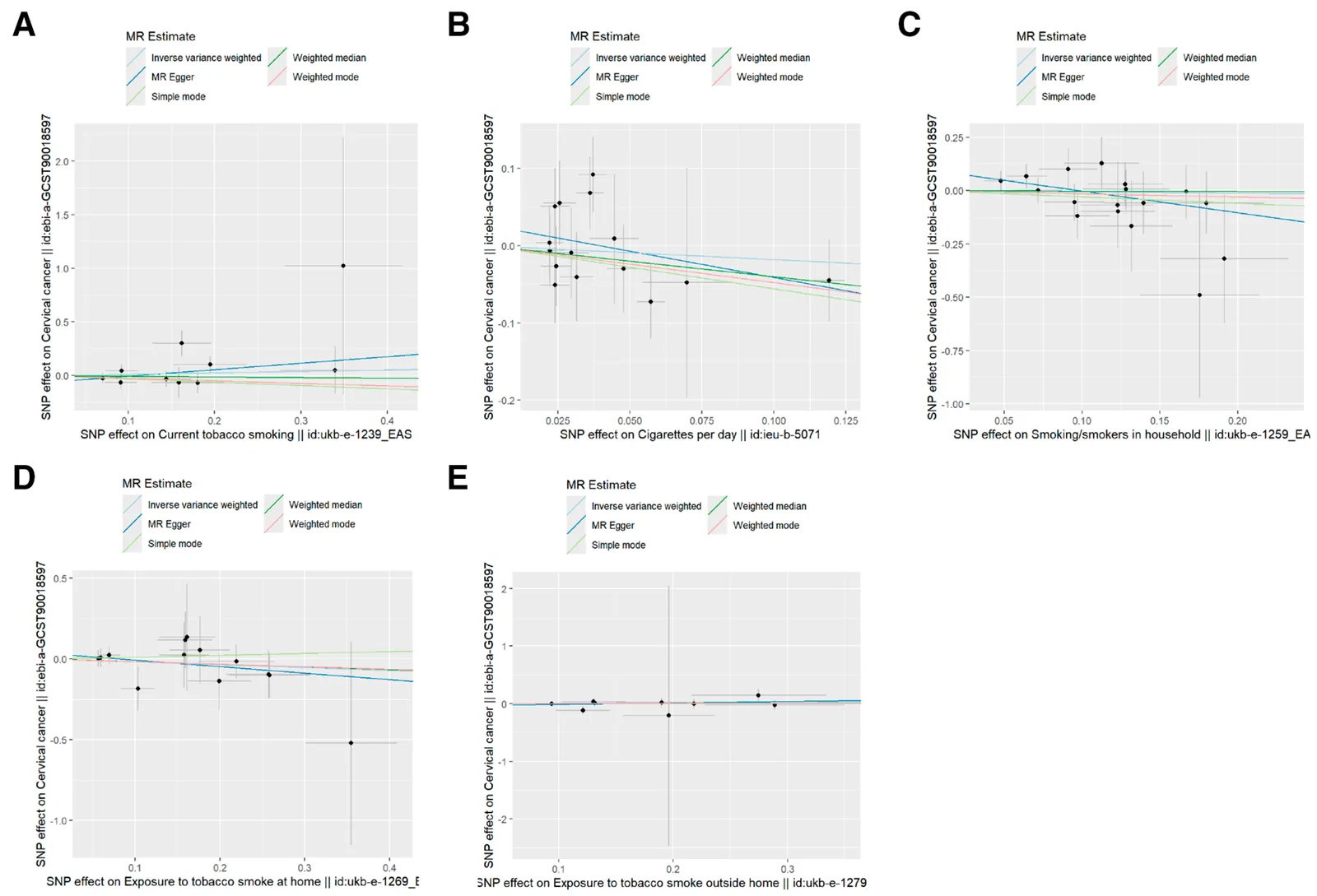
Mendelian Randomization intercept method analysis for horizontal pleiotropy. (A) Current tobacco smoking; (B) cigarettes per day; (C) smoking/smokers in household; (D) exposure to tobacco smoke at home; (E) exposure to tobacco smoke outside home. MR = Mendelian randomization, SNP = single-nucleotide polymorphism.

**Figure 4.**
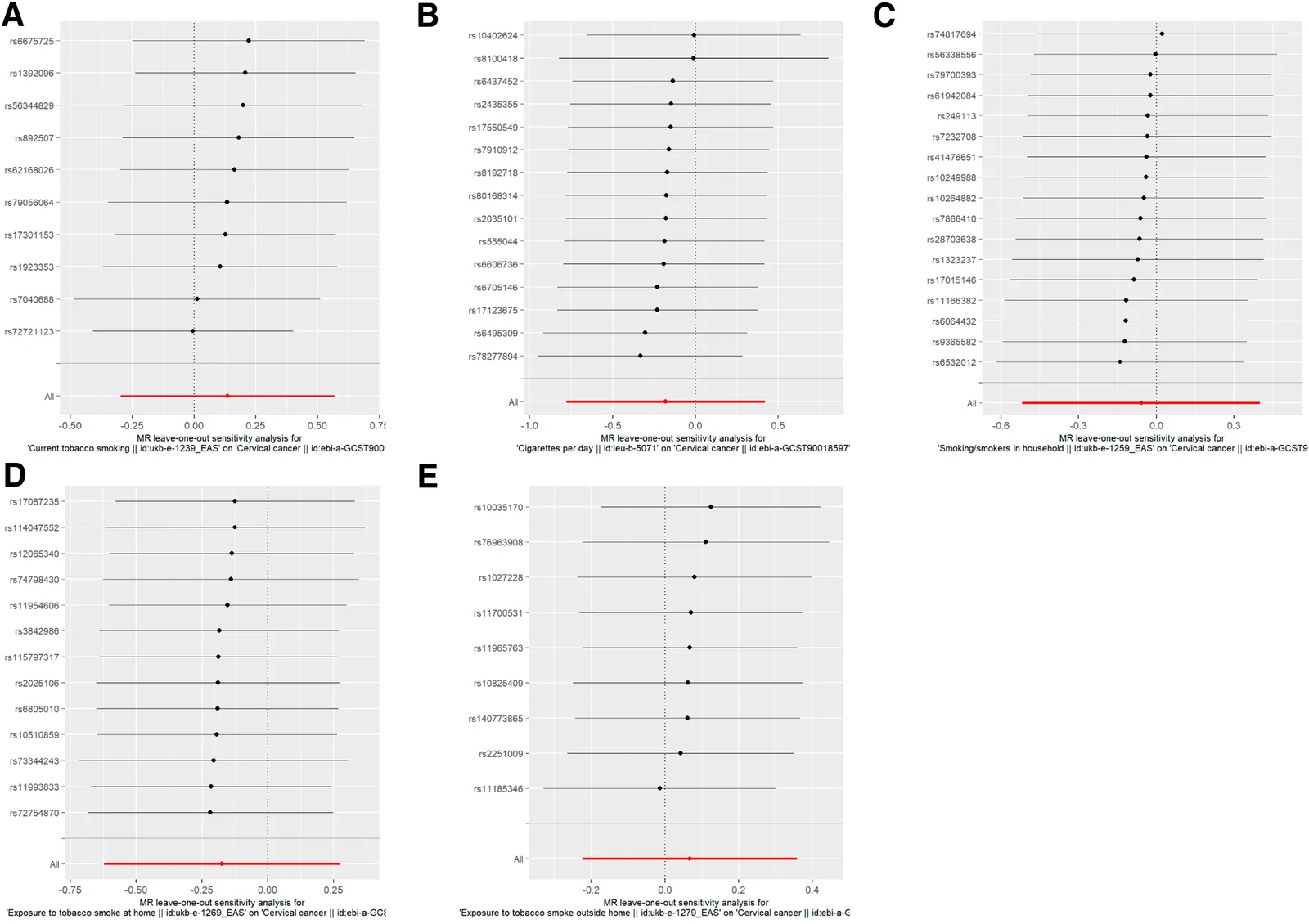
Leave-one-out analysis to assess the robustness of Mendelian randomization. (A) Current tobacco smoking; (B) cigarettes per day; (C) smoking/smokers in household; (D) exposure to tobacco smoke at home; (E) exposure to tobacco smoke outside home. MR = Mendelian randomization.

## 4. Discussion

This study systematically reveals the epidemiological burden and changing trends of CC in Asia, aiming to investigate the epidemiological characteristics of CC in Asian populations. Our GBD analysis indicates that, although the global incidence and DALYs of CC showed a downward trend from 1990 to 2021, Asia, particularly East Asia, remains a region with a substantial CC burden, even though the temporal trends varied across indicators and regions. Notably, China exhibited the most significant increase in the EAPC of prevalence, while South Korea was the only country in East Asia to show a declining trend, which may be attributed to its well-established national cancer screening program and high HPV vaccination coverage. Age-distribution analysis demonstrates that the highest burden of CC in terms of incidence, mortality, and DALYs occurs among women aged 55 to 59 years, highlighting the critical window for screening and intervention during midlife. Within the GBD attribution estimates, unsafe sexual behavior accounted for the largest reported proportion of attributable CC-related DALYs. However, our 2-sample MR analysis, using Japanese GWAS datasets, did not find statistically significant evidence supporting a causal relationship between 5 smoking-related exposures and CC risk after FDR correction.

The significant increase in the EAPC of CC prevalence in China is primarily attributable to the combined effect of a high HPV infection rate and the insufficient effectiveness of the screening system. Although high-risk HPV infections are widespread, screening coverage, particularly in rural and central/western regions, remains far below the level required for effective disease control, resulting in a large number of cases failing to be detected early.^[16,17]^ Simultaneously, the implementation of preventive measures has been delayed and uneven, with HPV vaccination yet to provide widespread protection.^[18]^ Compared to Western countries that have established systematic screening and high vaccine coverage,^[19]^ China’s CC prevention and control efforts are currently at a critical period, highlighting the urgency of accelerating vaccination efforts, expanding standardized screening, and narrowing healthcare resource disparities. It is noteworthy that Japan continues to face a high burden of CC. Firstly, there remains room for improvement in the coverage and participation rates of nationwide organized screening, with insufficient screening adherence in certain regions and population groups.^[20]^ Secondly, the HPV vaccination program was impacted by negative public perception in its early stages, leading to a sharp decline in coverage. Although vaccination rates have gradually recovered in recent years, this has resulted in an immunity gap within specific age cohorts.^[21]^ The combined effect of these factors results in a relatively prominent burden of CC in Japan compared to other developed countries with more successful prevention and control outcomes. Compared to other East Asian countries, South Korea has achieved notable success in CC prevention and control, with its incidence rate showing a consistent downward trend. Since 1999, South Korea has integrated CC screening into its national cancer prevention and control program, offering biennial free or heavily subsidized cytological examinations for women aged 20 and above.^[22]^ Additionally, through sustained public health education initiatives, active participation in screening and HPV vaccination among women has significantly improved in terms of both willingness and coverage.^[23]^ These practices demonstrate that well-established national policies and a robust healthcare system play a crucial role in the effective prevention and control of CC.

The age distribution of disease burden indicates that the peak of CC burden occurs in women aged 55 to 59, which aligns with the natural progression from high-risk HPV infection to invasive carcinoma and suggests a prolonged latency period.^[24]^ This finding underscores that middle-aged women (40–59 years) represent a key target population for prevention and control. Implementing intensified, precise screening and early intervention for this age group is a core strategy for reducing the overall incidence and mortality burden. Therefore, prevention and control programs should prioritize ensuring high screening coverage and follow‑up quality for this population to effectively halt disease progression. Insecure sexual behavior is considered a major risk factor for CC DALYs. Studies indicated that unsafe sexual behaviors (typically referring to multiple sexual partners, unprotected sex, and early initiation of sexual activity) were core behavioral risk factors leading to HPV infection and subsequent progression to CC.^[25]^ Epidemiological evidence consistently indicated that HPV infection is extremely common among sexually active women over their lifetime.^[26]^ This not only elevates the likelihood of exposure to high-risk HPV subtypes (e.g., HPV 16,18) but may also result in repeated infections with multiple HPV subtypes, thereby accumulating carcinogenic risks.^[27]^ Therefore, unsafe sexual behaviors represent a key behavioral intervention point for controllable CC. Promoting safe sex education can effectively block the occurrence of CC at the source of transmission.

Regarding smoking, our GBD finding of a substantial attributable fraction in Japan (18.97%) aligns with several large-scale observational studies. A systematic review and meta-analysis found that smoking increases the risk of cervical intraepithelial neoplasia and CC.^[28]^ Biological plausibility is strong, with tobacco carcinogens detected in cervical mucus and smoking shown to induce local immunosuppression, impairing clearance of HPV infection.^[29,30]^ However, Mendelian randomization analysis based on East Asian populations did not confirm a genetic causal relationship between smoking behavior and CC. This may be related to interference from other insufficiently measured confounding factors, such as sexual behavior patterns and socioeconomic status. Moreover, smoking may not be an independent pathogenic factor but rather acts as a cofactor, interacting with high-risk HPV infection to jointly promote carcinogenic progression.^[31]^ Nonetheless, based on the consistency of observational evidence and established biological mechanisms, promoting smoking cessation remains an important public health practice for protecting women’s health and reducing overall cancer risk.

Our study has several limitations. First, the GWAS data for both exposures and outcomes were primarily from Japanese populations, which may not fully represent the genetic diversity across Asia. Second, the CC case count in the GWAS was modest, limiting statistical power to detect anything but large causal effects. Third, the smoking behavior variables were self-reported and may not capture all aspects of tobacco exposure.

In summary, this study systematically delineates the epidemiological landscape of CC in Asia and highlights the particularly heavy disease burden borne by East Asia. Although GBD analyses and observational studies both identify smoking as a key risk factor, MR analysis based on East Asian populations did not confirm a causal relationship between the two. Future research requires larger GWAS sample sizes, more precise exposure measurement methods (such as biomarkers), and cross-ethnic MR analyses to further clarify the role of smoking in cervical carcinogenesis. It is particularly imperative in high-risk countries and regions across Asia to establish a population-wide screening system while vigorously promoting HPV vaccination and ensuring equitable access to healthcare resources.

## Author contributions

**Conceptualization:** Bing Wu, Qin Liu.

**Data curation:** Bing Wu.

**Formal analysis:** Bing Wu.

**Software:** Bing Wu.

**Supervision:** Qin Liu.

**Visualization:** Bing Wu.

**Writing – original draft:** Bing Wu, Qin Liu.

**Writing – review & editing:** Qin Liu.
